# (*Z*)-3-Hydrazinyl­idene-1-phenyl­indolin-2-one

**DOI:** 10.1107/S1600536810043916

**Published:** 2010-10-31

**Authors:** Hatem A. Abdel-Aziz, Ahmed Bari, T. Aboul-Fadl, Seik Weng Ng

**Affiliations:** a Department of Pharmaceutical Chemistry, College of Pharmacy, King Saud University, Riyadh 11451, Saudi Arabia; b Department of Chemistry, University of Malaya, 50603 Kuala Lumpur, Malaysia

## Abstract

The indoline fused-ring system of the title Schiff base, C_14_H_11_N_3_O, is planar (r.m.s. deviation = 0.005 Å); the phenyl substituent is aligned at 66.5 (1)° with respect to the ring system. The amino –NH_2_ unit forms an intra­molecular hydrogen bond with the carbonyl O atom. Mol­ecules are connected by an inter­molecular N—H⋯N hydrogen bond, generating a zigzag chain that runs along the short *c* axis of the unit cell.

## Related literature

For the synthesis of the title compound, see: de Diesbach & Heppner (1949 ▶).
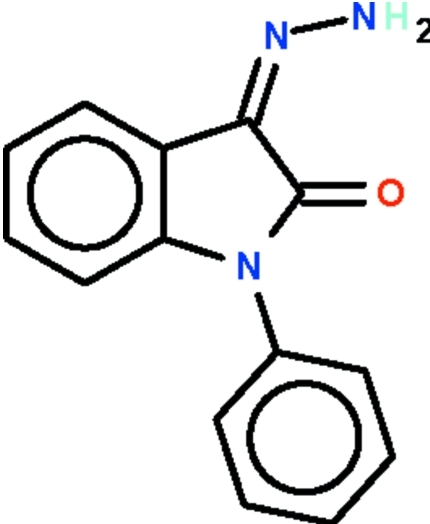


## Experimental

### 

#### Crystal data

C_14_H_11_N_3_O
*M* *_r_* = 237.26
Orthorhombic, 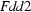

*a* = 19.328 (3) Å
*b* = 41.612 (5) Å
*c* = 5.6288 (7) Å
*V* = 4527 (1) Å^3^
*Z* = 16
Mo *K*α radiation
μ = 0.09 mm^−1^
*T* = 100 K
0.35 × 0.04 × 0.02 mm

#### Data collection

Bruker SMART APEX diffractometer
10569 measured reflections
1430 independent reflections
1188 reflections with *I* > 2σ(*I*)
*R*_int_ = 0.079

#### Refinement

*R*[*F* ^2^ > 2σ(*F* ^2^)] = 0.040
*wR*(*F* ^2^) = 0.092
*S* = 1.03
1430 reflections
171 parameters
3 restraints
H atoms treated by a mixture of independent and constrained refinement
Δρ_max_ = 0.27 e Å^−3^
Δρ_min_ = −0.22 e Å^−3^
Absolute structure: 1138 Friedel pairs were merged

### 

Data collection: *APEX2* (Bruker, 2009 ▶); cell refinement: *SAINT* (Bruker, 2009 ▶); data reduction: *SAINT*; program(s) used to solve structure: *SHELXS97* (Sheldrick, 2008 ▶); program(s) used to refine structure: *SHELXL97* (Sheldrick, 2008 ▶); molecular graphics: *X-SEED* (Barbour, 2001 ▶); software used to prepare material for publication: *publCIF* (Westrip, 2010 ▶).

## Supplementary Material

Crystal structure: contains datablocks global, I. DOI: 10.1107/S1600536810043916/bt5393sup1.cif

Structure factors: contains datablocks I. DOI: 10.1107/S1600536810043916/bt5393Isup2.hkl

Additional supplementary materials:  crystallographic information; 3D view; checkCIF report

## Figures and Tables

**Table 1:** Hydrogen-bond geometry (Å, °)

*D*—H⋯*A*	*D*—H	H⋯*A*	*D*⋯*A*	*D*—H⋯*A*
N3—H1⋯O1	0.88 (1)	2.06 (2)	2.772 (3)	137 (3)
N3—H2⋯N2^i^	0.89 (1)	2.22 (1)	3.102 (3)	177 (3)

Symmetry code: (i) 
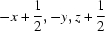
.

**Fig. 1.**
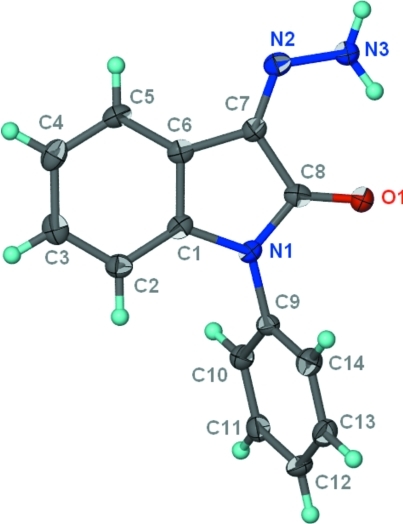
Anisotropic displacement ellipsoid plot (Barbour, 2001) of C14H11N3O at the 70% probability level; H atoms are drawn as spheres of arbitrary radius.

**Fig. 2.**
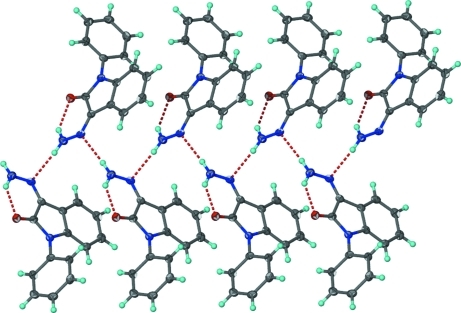
Hydrogen-bonded chain structure.

## supplementary crystallographic information

### Comment

Isatin derivatives such as phenylisatin have been studied in the context of its biological properties. We have synthesized the condensation product of phenylisatin with hydrazine for evaluation as a chemotherapeutic agent. The title hydrazone (Scheme I) is only mentioned once in the chemical literature (de Diesbach & Heppner, 1949). The indolinyl fused-ring is twisted with respect to the phenyl substituent by 66.5 (1)° (Fig. 1). The amino –NH2 unit forms an intramolecular hydrogen bond with the carbonyl O atom; the unit uses its other H atom for intermolecular hydrogen bonding to the two-coordinate N atom (Fig. 2). The intramolecular N—H···N interaction generates a zigzag chain that runs along the short *c* axis of the unit cell.

### Experimental

1-Phenylindoline-2,3-dione (0.220 g, 1 mmol) and hydrazine hydrate (0.055 g, 1.1 mmol) were dissolved in methanol (25 ml) and the solution heated for 1 h. The solvent was evaporated and the product recrystallized from ethanol to give yellow prismatic crystals; yield 70%.

### Refinement

Carbon-bound H atoms were placed in calculated positions (C—H 0.95 Å) and were included in the refinement in the riding model approximation, with *U*_iso_(H) set to 1.2 times *U*_eq_(C).

The amino H atoms were located in a difference Fourier map, and were refined isotropically with a distance restraint of N—H 0.88 (1) Å.

As the structure has no anomalous scatterer, 1138 Friedel pairs were merged.

### Crystal data

**Table:**

C_14_H_11_N_3_O	*F*(000) = 1984
*M**_r_* = 237.26	*D*_x_ = 1.392 Mg m^−^^3^
Orthorhombic, *F**d**d*2	Mo *K*α radiation, λ = 0.71073 Å
Hall symbol: F 2 -2d	Cell parameters from 1516 reflections
*a* = 19.328 (3) Å	θ = 2.3–27.8°
*b* = 41.612 (5) Å	µ = 0.09 mm^−^^1^
*c* = 5.6288 (7) Å	*T* = 100 K
*V* = 4527 (1) Å^3^	Prism, yellow
*Z* = 16	0.35 × 0.04 × 0.02 mm

### Data collection

**Table:**

Bruker SMART APEX diffractometer	1188 reflections with *I* > 2σ(*I*)
Radiation source: fine-focus sealed tube	*R*_int_ = 0.079
graphite	θ_max_ = 27.5°, θ_min_ = 2.3°
ω scans	*h* = −24→24
10569 measured reflections	*k* = −54→54
1430 independent reflections	*l* = −7→7

### Refinement

**Table:**

Refinement on *F*^2^	Secondary atom site location: difference Fourier map
Least-squares matrix: full	Hydrogen site location: inferred from neighbouring sites
*R*[*F*^2^ > 2σ(*F*^2^)] = 0.040	H atoms treated by a mixture of independent and constrained refinement
*wR*(*F*^2^) = 0.092	*w* = 1/[σ^2^(*F*_o_^2^) + (0.054*P*)^2^] where *P* = (*F*_o_^2^ + 2*F*_c_^2^)/3
*S* = 1.03	(Δ/σ)_max_ = 0.001
1430 reflections	Δρ_max_ = 0.27 e Å^−^^3^
171 parameters	Δρ_min_ = −0.22 e Å^−^^3^
3 restraints	Absolute structure: 1138 Friedel pairs were merged
Primary atom site location: structure-invariant direct methods	

### Fractional atomic coordinates and isotropic or equivalent isotropic displacement parameters (Å^2^)

**Table:**

	*x*	*y*	*z*	*U*_iso_*/*U*_eq_	
O1	0.27334 (9)	0.09714 (4)	0.4984 (3)	0.0193 (4)	
N1	0.34809 (10)	0.10293 (4)	0.1772 (4)	0.0161 (5)	
N2	0.28770 (10)	0.02682 (5)	0.3752 (4)	0.0177 (5)	
N3	0.24626 (11)	0.03226 (5)	0.5601 (4)	0.0201 (5)	
C1	0.37995 (12)	0.08200 (6)	0.0120 (5)	0.0158 (6)	
C2	0.42392 (12)	0.08987 (6)	−0.1705 (5)	0.0179 (6)	
H2A	0.4368	0.1115	−0.2002	0.022*	
C3	0.44890 (12)	0.06483 (6)	−0.3103 (5)	0.0206 (6)	
H3	0.4788	0.0695	−0.4396	0.025*	
C4	0.43087 (13)	0.03306 (6)	−0.2640 (5)	0.0213 (6)	
H4	0.4485	0.0164	−0.3621	0.026*	
C5	0.38720 (12)	0.02553 (6)	−0.0752 (5)	0.0192 (6)	
H5	0.3753	0.0038	−0.0428	0.023*	
C6	0.36135 (12)	0.05005 (6)	0.0645 (5)	0.0160 (5)	
C7	0.31593 (12)	0.05137 (6)	0.2694 (4)	0.0158 (6)	
C8	0.30841 (12)	0.08581 (6)	0.3372 (5)	0.0162 (5)	
C9	0.35079 (12)	0.13736 (5)	0.1607 (5)	0.0158 (5)	
C10	0.32204 (13)	0.15233 (6)	−0.0360 (5)	0.0181 (5)	
H10	0.3004	0.1400	−0.1570	0.022*	
C11	0.32528 (13)	0.18547 (6)	−0.0538 (5)	0.0207 (6)	
H11	0.3055	0.1960	−0.1872	0.025*	
C12	0.35737 (13)	0.20340 (6)	0.1227 (5)	0.0210 (6)	
H12	0.3597	0.2261	0.1097	0.025*	
C13	0.38601 (14)	0.18805 (6)	0.3177 (5)	0.0217 (6)	
H13	0.4078	0.2004	0.4386	0.026*	
C14	0.38314 (13)	0.15489 (6)	0.3378 (5)	0.0198 (6)	
H14	0.4031	0.1444	0.4710	0.024*	
H1	0.2435 (15)	0.0522 (4)	0.612 (6)	0.031 (8)*	
H2	0.2355 (14)	0.0151 (5)	0.646 (5)	0.036 (9)*	

### Atomic displacement parameters (Å^2^)

**Table:**

	*U*^11^	*U*^22^	*U*^33^	*U*^12^	*U*^13^	*U*^23^
O1	0.0229 (9)	0.0166 (8)	0.0185 (11)	0.0001 (7)	0.0040 (9)	−0.0017 (8)
N1	0.0184 (10)	0.0129 (9)	0.0170 (12)	−0.0001 (8)	0.0014 (9)	−0.0021 (9)
N2	0.0164 (10)	0.0159 (10)	0.0207 (13)	−0.0012 (8)	−0.0024 (10)	−0.0009 (9)
N3	0.0242 (11)	0.0158 (11)	0.0203 (13)	−0.0006 (9)	0.0045 (11)	−0.0002 (10)
C1	0.0153 (11)	0.0148 (11)	0.0173 (15)	0.0024 (9)	−0.0039 (11)	−0.0006 (10)
C2	0.0176 (12)	0.0160 (11)	0.0202 (14)	0.0004 (9)	−0.0008 (12)	0.0017 (11)
C3	0.0150 (11)	0.0274 (13)	0.0194 (14)	0.0013 (10)	−0.0011 (12)	0.0005 (12)
C4	0.0183 (12)	0.0227 (12)	0.0229 (16)	0.0056 (10)	−0.0027 (12)	−0.0055 (11)
C5	0.0206 (12)	0.0138 (11)	0.0232 (15)	−0.0005 (9)	−0.0026 (13)	−0.0018 (11)
C6	0.0137 (11)	0.0163 (11)	0.0179 (14)	0.0000 (9)	−0.0036 (11)	0.0000 (11)
C7	0.0145 (12)	0.0154 (12)	0.0175 (15)	0.0002 (9)	−0.0020 (11)	−0.0014 (10)
C8	0.0147 (11)	0.0155 (11)	0.0183 (14)	−0.0006 (9)	−0.0028 (11)	0.0025 (11)
C9	0.0157 (12)	0.0125 (11)	0.0192 (14)	0.0003 (9)	0.0033 (10)	0.0003 (11)
C10	0.0211 (12)	0.0175 (12)	0.0159 (14)	−0.0013 (10)	0.0003 (11)	−0.0013 (11)
C11	0.0246 (13)	0.0204 (12)	0.0172 (14)	0.0034 (10)	0.0039 (12)	0.0011 (11)
C12	0.0246 (13)	0.0115 (10)	0.0268 (16)	−0.0012 (10)	0.0058 (13)	−0.0023 (11)
C13	0.0213 (13)	0.0216 (12)	0.0222 (15)	−0.0037 (10)	0.0025 (12)	−0.0066 (12)
C14	0.0202 (12)	0.0212 (12)	0.0181 (14)	0.0001 (10)	−0.0020 (12)	−0.0016 (11)

### Geometric parameters (Å, °)

**Table:**

O1—C8	1.227 (3)	C5—C6	1.381 (4)
N1—C8	1.381 (3)	C5—H5	0.9500
N1—C1	1.415 (3)	C6—C7	1.451 (3)
N1—C9	1.436 (3)	C7—C8	1.491 (3)
N2—C7	1.302 (3)	C9—C14	1.385 (4)
N2—N3	1.332 (3)	C9—C10	1.387 (4)
N3—H1	0.883 (10)	C10—C11	1.384 (3)
N3—H2	0.888 (10)	C10—H10	0.9500
C1—C2	1.373 (4)	C11—C12	1.388 (4)
C1—C6	1.408 (3)	C11—H11	0.9500
C2—C3	1.392 (4)	C12—C13	1.386 (4)
C2—H2A	0.9500	C12—H12	0.9500
C3—C4	1.392 (4)	C13—C14	1.385 (3)
C3—H3	0.9500	C13—H13	0.9500
C4—C5	1.393 (4)	C14—H14	0.9500
C4—H4	0.9500		
			
C8—N1—C1	110.66 (19)	N2—C7—C6	126.0 (2)
C8—N1—C9	125.2 (2)	N2—C7—C8	126.6 (2)
C1—N1—C9	123.8 (2)	C6—C7—C8	107.4 (2)
C7—N2—N3	118.4 (2)	O1—C8—N1	126.3 (2)
N2—N3—H1	117 (2)	O1—C8—C7	127.8 (2)
N2—N3—H2	115 (2)	N1—C8—C7	105.9 (2)
H1—N3—H2	124 (3)	C14—C9—C10	121.3 (2)
C2—C1—C6	122.7 (2)	C14—C9—N1	119.7 (2)
C2—C1—N1	127.9 (2)	C10—C9—N1	119.0 (2)
C6—C1—N1	109.4 (2)	C11—C10—C9	119.2 (2)
C1—C2—C3	117.3 (2)	C11—C10—H10	120.4
C1—C2—H2A	121.3	C9—C10—H10	120.4
C3—C2—H2A	121.3	C10—C11—C12	120.3 (3)
C4—C3—C2	121.2 (3)	C10—C11—H11	119.9
C4—C3—H3	119.4	C12—C11—H11	119.9
C2—C3—H3	119.4	C11—C12—C13	119.8 (2)
C5—C4—C3	120.5 (2)	C11—C12—H12	120.1
C5—C4—H4	119.7	C13—C12—H12	120.1
C3—C4—H4	119.7	C14—C13—C12	120.5 (3)
C6—C5—C4	119.2 (2)	C14—C13—H13	119.7
C6—C5—H5	120.4	C12—C13—H13	119.7
C4—C5—H5	120.4	C9—C14—C13	119.0 (3)
C5—C6—C1	119.0 (2)	C9—C14—H14	120.5
C5—C6—C7	134.4 (2)	C13—C14—H14	120.5
C1—C6—C7	106.6 (2)		
			
C8—N1—C1—C2	−178.8 (2)	C1—N1—C8—O1	−178.4 (2)
C9—N1—C1—C2	7.9 (4)	C9—N1—C8—O1	−5.2 (4)
C8—N1—C1—C6	−0.4 (3)	C1—N1—C8—C7	0.3 (3)
C9—N1—C1—C6	−173.7 (2)	C9—N1—C8—C7	173.5 (2)
C6—C1—C2—C3	1.7 (4)	N2—C7—C8—O1	−2.1 (4)
N1—C1—C2—C3	179.9 (2)	C6—C7—C8—O1	178.5 (2)
C1—C2—C3—C4	−1.1 (4)	N2—C7—C8—N1	179.2 (2)
C2—C3—C4—C5	−0.1 (4)	C6—C7—C8—N1	−0.1 (2)
C3—C4—C5—C6	0.7 (4)	C8—N1—C9—C14	70.8 (3)
C4—C5—C6—C1	0.0 (4)	C1—N1—C9—C14	−116.8 (3)
C4—C5—C6—C7	179.9 (2)	C8—N1—C9—C10	−110.5 (3)
C2—C1—C6—C5	−1.2 (4)	C1—N1—C9—C10	61.8 (3)
N1—C1—C6—C5	−179.7 (2)	C14—C9—C10—C11	−0.6 (4)
C2—C1—C6—C7	178.8 (2)	N1—C9—C10—C11	−179.2 (2)
N1—C1—C6—C7	0.3 (3)	C9—C10—C11—C12	0.4 (4)
N3—N2—C7—C6	−179.1 (2)	C10—C11—C12—C13	−0.3 (4)
N3—N2—C7—C8	1.8 (4)	C11—C12—C13—C14	0.3 (4)
C5—C6—C7—N2	0.6 (4)	C10—C9—C14—C13	0.6 (4)
C1—C6—C7—N2	−179.4 (2)	N1—C9—C14—C13	179.2 (2)
C5—C6—C7—C8	179.9 (3)	C12—C13—C14—C9	−0.4 (4)
C1—C6—C7—C8	−0.1 (2)		

### Hydrogen-bond geometry (Å, °)

**Table:**

*D*—H···*A*	*D*—H	H···*A*	*D*···*A*	*D*—H···*A*
N3—H1···O1	0.88 (1)	2.06 (2)	2.772 (3)	137 (3)
N3—H2···N2^i^	0.89 (1)	2.22 (1)	3.102 (3)	177 (3)

Symmetry codes: (i) −*x*+1/2, −*y*, *z*+1/2.
